# The Association Between FokI Vitamin D Receptor Polymorphisms With Metabolic Syndrome Among Pregnant Arab Women

**DOI:** 10.3389/fendo.2022.844472

**Published:** 2022-02-24

**Authors:** Maysa Alzaim, Nasser M. Al-Daghri, Shaun Sabico, Mona A. Fouda, Sara Al-Musharaf, Malak N. K. Khattak, Abdul Khader Mohammed, Abdulrahman Al-Ajlan, Dalal N. Binjawhar, Richard Wood

**Affiliations:** ^1^ Nutrition Department School of Public Health & Health Sciences, University of Massachusetts, Amherst, MA, United States; ^2^ Biochemistry Department, College of Science, King Saud University, Riyadh, Saudi Arabia; ^3^ Endocrinology Division, Department of Medicine, College of Medicine, King Saud University, Riyadh, Saudi Arabia; ^4^ Department of Community Health, College of Applied Medical Science, King Saud University, Riyadh, Saudi Arabia; ^5^ Sharjah Institute for Medical Research, University of Sharjah, Sharjah, United Arab Emirates; ^6^ Department of Clinical Lab Sciences, College of Applied Medical Sciences, King Saud University, Riyadh, Saudi Arabia; ^7^ Department of Chemistry, College of Science, Princess Noura bint Abdulrahman University, Riyadh, Saudi Arabia

**Keywords:** metabolic syndrome, vitamin D polymorphisms, pregnancy, gestational diabetes mellitus, genetic marker

## Abstract

Metabolic syndrome (MetS) is a serious health condition that is becoming extremely threatening in Saudi Arabia. The link between vitamin D receptor (VDR) gene polymorphisms and maternal MetS has been observed in several ethnic groups, but is yet to be clarified in the Arabian population. This study aims to investigate the relationship between the *FokI* VDR genotype and the risk of MetS and its components in pregnant Saudi women. A cross-sectional study was conducted using 368 pregnant Saudi women on first trimester screened for MetS (44 with MetS and 324 without MetS). Measurements included anthropometrics, glycemic and lipid profile and 25(OH)D. TaqMan genotyping assay was used to determine *Fokl* VDR genotype of participants. Vitamin D deficiency (25(OH)D <50nmol/l) was seen in 85% of the participants. An estimated 12% of participants had MetS. In the MetS group, the *FokI* VDR genotyping frequencies for *FF, Ff*, and *ff* genotypes were 50%, 36.4% and 13.6%, respectively. In controls, the frequencies were 62.7%, 31.4% and 5.9%, respectively. No significant association between the individual MetS components and *FokI* VDR genotypes were observed. Nevertheless, carriers of the *ff* allele had a significant risk for full maternal MetS [Odds Ratio 4.2 (95% Confidence Interval 1.4-12.2; adjusted *p*=0.009). The study suggests that the *ff FokI* VDR genotype is a genetic marker of maternal MetS in pregnant Arabian women. Prospective studies that include neonatal outcomes may confirm present findings.

## Introduction

Metabolic syndrome (MetS) is a cluster of conditions that is present when three or more of the following five characteristics are met: central obesity, high cholesterol, elevated blood pressure, dyslipidemia, and elevated fasting blood glucose (1). MetS is a well-established independent risk factor for type 2 diabetes mellitus (T2DM) and cardiovascular disease (CVD) (2). The prevalence of MetS is increasing globally, rising from a rate of 20% among adult populations to 40% (3). Almost 25% of the adult population in the United States suffers from MetS (4), but still lower as compared to Saudi Arabia, where 35% of the adult population has full MetS (5). Among at risk populations, women with MetS are at a higher risk of developing maternal complications like preeclampsia and coma, in addition to experiencing adverse perinatal effects like preterm birth, jaundice, neonatal macrosomia, and malformations (1, 4). The presence of MetS during pregnancy alone increases maternal CVD by as much as seven-fold (6), with offspring of high-risk pregnancies also having a higher risk of developing CVD, MetS, and T2DM (1, 7). The Riyadh Mother and Baby Study (RAHMA), a large cohort study in Saudi Arabia, revealed that more than 68% of the 14,568 pregnant women studied were either overweight or obese, while 24% had developed gestational diabetes mellitus (GDM), considered among the highest in the world (8). Aside from MetS and its components as GDM risk factors (9), an emerging GDM risk factor considered unique in the Arabian population is vitamin D deficiency (10).

On the physiological level, 1.25-dihydroxyvitamin D is the active form of vitamin D, and its activity is mediated *via* the cellular vitamin D nuclear receptor (VDR) protein (11). VDR is abundantly expressed in most body tissues, including uteroplacental parts, and is important in bone metabolism, calcium homeostasis and other non-calcemic functions (12, 13). In normal pregnancy, VDRs can regulate implantation as well as hormonal and immune modulations in the placenta (14). Similar to the nature of most nuclear receptors, the full function of VDR can be altered by several single nucleotide polymorphisms (SNPs), including the *FokI* VDR gene polymorphism (15).

The *FokI* VDR gene polymorphism is of particular interest, given that it occurs within the transcription start codon (11). Furthermore, its polymorphic form (*f*) shifts the start codon position, meaning that the expression of the VDR protein is three amino acids longer than the wild-type (*F*) allele (11). The shorter-wildtype version of the VDR protein is more active than the longer VDR protein. In some epidemiologic studies, the shorter *FokI* VDR allelic variation has been associated with a host of adverse disease outcomes (16, 17), including increased risk to MetS in adults (2, 3, 15, 16, 18, 19). Furthermore, increased susceptibility to GDM from expectant mothers having the *FokI* variant were observed among expectant mothers coming from Turkey (20) and Iran (21), but not Chinese (22) and Saudi Arabians (23), which means that *FokI* may confer susceptibility only to select populations (24). To date, there is scarcity of evidence linking MetS and *FokI* VDR SNPs in pregnant women, more so in understudied ethnic groups such as Saudi Arabians. Since vitamin D deficiency is endemic in the Saudi population, this group is susceptible to the adverse effects of vitamin D-related disorders, given the presence of the relatively ineffective longer VDR protein found in women with the *ff* VDR genotype. This study is therefore intended to explore the link between VDR *FokI* genotypes and the risk of MetS and its components in Saudi Arabian pregnant women.

## Materials and Methods

### Study Design and Sample Population

This study comprises part of a larger prospective cohort study, “*Vitamin D and Pregnancy in Saudi Women*.” This cross-sectional study focuses on a subset of study participants—324 healthy pregnant women and 44 pregnant women with MetS—for a total of 368 participants. These women visited one of three Saudi hospitals in their second trimester of pregnancy (24–28 weeks) between December 2013 and January 2016: King Khaled University Hospital (KKUH), King Salman bin Abdulaziz Hospital, or King Fahad Medical City (KFMC), all in the capital city of Saudi Arabia, Riyadh. All of the necessary approvals were collected, meaning that this study has full ethical approval to collect samples and patient data. Approval was secured from the Ethics Committee of the College of Medicine (Approval No. E-13-1013, February 11, 2014) King Saud University, Riyadh. Each patient also provided his or her express written consent.

### Inclusion and Exclusion Criteria

The study focused on healthy Saudi women who were pregnant and aged between 18 and 40 years. These participants were enrolled in the study before 16 weeks of gestation and exhibited no previous history of DM. Participants were excluded if they were pregnant non-Saudis over 16 weeks age of gestation, those on vitamin D supplements, oral glucocorticoids, calcium, cardiac medications or any drug known to interfere with vitamin D, calcium absorption, or parathyroid disorders, those with malabsorption syndrome, hypertension and other pre-existing conditions including previous history of GDM.

### Anthropometric Measurements

Anthropometric measurements included height (cm) and weight (kg) in order to calculate BMI (kg/m^2^) as well as pre-pregnancy weight (kg) and pre-pregnancy BMI (kg/m^2^). Blood pressure information (mmHg) was also noted. Body weight measurements were taken without shoes, and the participants used lightweight clothing. This measurement was recorded to the nearest 0.1 kg (Digital Person Scale, ADAM Equipment Inc., USA). The pre-pregnancy weight was self-reported during the prenatal visit, and this was used to classify the patients according to the World Health Organization’s (WHO) BMI definitions: underweight: < 18.5 kg/m^2^; normal weight: 18.5–24.9 kg/m^2^; overweight: 25.0–29.9 kg/m^2^; or obese: ≥ 30.0 kg/m^2^ (25). The height of the participants was measured to the nearest 0.5 cm using the Digital Pearson Scale at the first pregnancy visit. During the measurement, patients were standing upright and without shoes. A mercurial sphygmomanometer was used to measure blood pressure (mmHg).

### Biochemical Assessment and MetS Screening

Participants were asked to fast for > 10 hours before fasting blood samples of 10ml were taken. These samples were collected with a sterile vacutainer blood collection apparatus. Samples were aliquoted and moved to a -80°C freezer. The Chair for Biomarkers of Chronic Diseases (CBCD) in King Saud University, Riyadh, Saudi Arabia was responsible for storing and analyzing the blood samples. Electro-chemiluminescence binding assay 2012 (ECLIA) (Roche Diagnostics GmbA, Mannheim, Germany) and commercially available IDS kits (IDS Ltd., Boldon Colliery, Tyne & Wear, UK) were used to measure serum 25(OH)D. The inter- and intra-assay coefficients of variation (CV) for 25(OH)D ELISA are 5.3% and 4.6%, respectively, with 100% cross-reactivity to 25(OH)D3 and 75% cross-reactivity to 25(OH)D2. According to national and regional guidelines, the cutoff values for vitamin D deficiency are less than 50 nmol/L for serum 25(OH)D and, for sufficiency serum 25(OH)D, more than 50nmol/L (26, 27).

A chemical analyzer (Konelab, Vantaa, Finland) was used to measure triglycerides (TG), fasting serum glucose (FBG) and lipid profile [including total cholesterol (TC)], high-density lipoprotein cholesterol (HDL-C), and low-density lipoprotein cholesterol (LDL-C) (28). Point-of-care (POC) devices (Accu-Check Active, Roche Diagnostics, Mannheim, Germany) were used to measure the HbA_1C_ from the whole blood.

**Figure 1:**
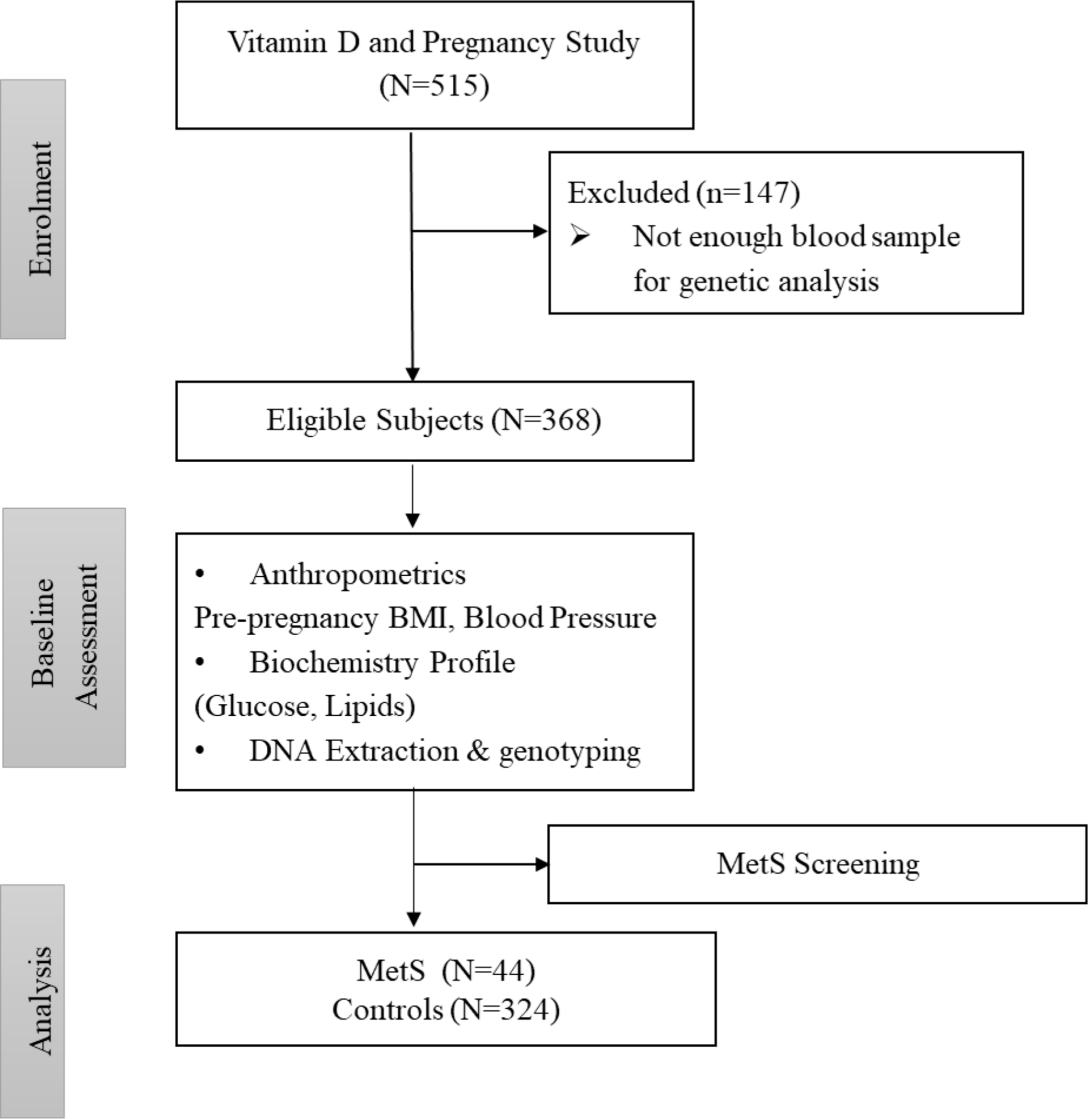
Flowchart of participants.

Under the definition of MetS proposed for the obstetric population, a pregnant woman can be defined as having MetS if she has three or more of these risk factors: a pre-pregnancy body mass index (BMI) > 30 kg/m^2^; level of serum triglycerides (TG) ≥ 1.7mmol/L; serum high-density lipoprotein (HDL)-cholesterol level < 1.3mmol/L; fasting serum glucose level ≥ 5.6mmol/L, and blood pressure level ≥ 130/85 mmHg (10). The participants who fell outside of this definition were grouped as controls. **Figure 1** shows the flowchart of participants.

### DNA Extraction and Quantification

For extracting Genomic DNC from the whole blood, innuPREP mini blood kits (Analytik Jena, Germany) were used according to the instructions of the manufacturer. In brief, a 1.5 ml reaction tube containing lysis buffer and Proteinase K hosted 200 µl of a whole blood sample. This was mixed using vortex and incubated for 10 minutes at 60°C. An appropriate quantity of binding solution was also added before the tube itself was transferred to a spin filter column and centrifuged for a full minute at 12,000 rpm. The washing of the DNA was performed using washing solution C—included with the kit—followed by washing solution BS. For the final step, the spin filter was inserted into a 1.5 m elution tube while 200 µl of elution buffer was pre-warmed and added. Once centrifugation was completed, the DNA was saved for further analysis at a temperature of -20°C. A Nano-Drop spectrophotometer was used to determine the concentration and purity (260/280) of the DNA (29).

### *FOK-I* SNP Genotyping

The *Fok-I* SNP (rs 2228570) was assessed using a pre-designed TaqMan genotyping assay from Applied Biosystems, Foster City, CA, USA (assay ID: C_12060045_20). Amplification reactions were performed in a volume of 10 µL containing 1X TaqMan genotyping Master Mix (Applied Biosystems), 1X mix of unlabeled PCR primers and TaqMan MGB probes, and 30 ng of template DNA. All amplification and detection was conducted in 96-well PCR plates using a Bio-Rad CFX96 Real-Time PCR Detection System (Bio-Rad, Milan, Italy). Thermal cycling was initiated with a denaturation step of 10 min at 95°C, followed by 45 cycles of 15 s at 95°C and 90 s at 60°C. After PCR was completed, allelic discrimination was analyzed using the Bio-Rad CFX Manager Software (Version 1.6, Bio-Rad). Genotype assignment was determined by plotting the end point relative fluorescent units (RFU) for one fluorophore (allele 1 on the x-axis) against the RFU for the other fluorophore (allele 2 on the y-axis) on the allelic discrimination. All PCR reactions were set up in a dedicated PCR area with dedicated PCR pipettes and reagents.

### Sample Size and Statistical Analysis

Based on the association between HDL-cholesterol and FoKI polymorphism reported in previous literature with the effect size of 0.162 (17), the required sample size for this study at 95% and 80% power is N=366. Data were analyzed using SPSS (version 21.0, IBM). Continuous data are presented as mean ± standard deviation (SD) for Gaussian variables, and non-Gaussian variables are presented as median (25^th^ and 75^th^) percentiles. Categorical data are presented as frequencies and percentages (%). All continuous variables were checked for normality using a Kolmogorov-Smirnov test. Non-Gaussian variables were log-transformed prior to parametric analysis. Independent T-tests, analysis of variance (ANOVA), and Mann-Whitney and Kruskal-Wallis H tests were used to compare mean differences in Gaussian and non-Gaussian variables. Odds-ratio and X^2^ tests were done using binary logistics and multinomial logistic regression. A *P*-value < 0.05 was considered statistically significant.

## Results

### General Characteristics and Components of MetS

Participant characteristics are presented in **Table 1**. The prevalence of MetS in our study was 12%. The MetS group in our study was significantly older and had a higher pre-pregnancy BMI than the controls (*p* < 0.02 and < 0.01, respectively). There was no significant difference in circulating 25(OH)D levels. Compared with the controls, TG, insulin, HbA_1C_, fasting serum glucose, HOMA-IR, and diastolic blood pressure were significantly higher in the MetS group (*p* < 0.01 for all variables), while HDL cholesterol was found to be significantly lower (1.6 ± 0.4 vs. 1.3 ± 0.5 mg/dl, *p* < 0.01).

**Table 1:** Demographic and biochemical characteristics of MetS vs. control groups.

Parameters	All	MetS	Control	*P-*value	*P*-value*
N	368	44 (12.0)	324 (88.0)		
Age (years)	29.1 ± 5.6	31.3 ± 6.9	28.7 ± 5.3	0.02	
Pre-pregnancy BMI (kg/m^2^)	26.9 ± 5.9	33.3 ± 4.1	25.9 ± 5.5	<0.001	
Current BMI (kg/m^2^)	28.2 ± 6.2	34.7 ± 4.5	27.5 ± 5.9	<0.001	0.96
Parity	2 (1–4)	2 (1–5)	2 (1–4)	0.21	0.54
Systolic Blood Pressure (mmHg)	113.3 ± 12.9	119.1 ± 15.7	112.3 ± 12.1	0.02	0.27
Diastolic Blood Pressure (mmHg)	66.9 ± 9.2	71.7 ± 11.4	66.1 ± 8.5	0.010	0.03
Hba1c (%)	4.8 ± 0.5	5.1 ± 0.7	4.8 ± 0.8	<0.001	0.005
Fasting Glucose (mmol/L)	4.6 ± 1.0	5.6 ± 1.5	4.5 ± 0.8	<0.001	<0.001
HOMA-IR	1.5 (0.9-2.5)	3.1 (1.7-5.2)	1.3 (0.9-2.3)	<0.001	<0.001
Insulin (uU/ml)	7.5 (4.5-13.1)	14.2 (7.9-22.8)	6.9 (4.1-11.9)	<0.001	0.003
HDL-Cholesterol (mmol/L)	1.5 ± 0.4	1.3 ± 0.5	1.6 ± 0.4	<0.001	<0.001
LDL-Cholesterol (mmol/L)	3.9 ± 1.3	3.9 ± 1.4	3.9 ± 1.3	0.70	0.27
Total Cholesterol (mmol/L)	6.2 ± 1.2	6.4 ± 1.3	6.2 ± 1.5	0.27	0.08
Triglycerides (mmol/L)	1.8 (1.4-2.3)	2.6 (2.1-3.4)	1.7 (1.3-2.2)	<0.001	<0.001
25(OH)D (nmol/L)	33.4 (21.3-53.7)	29.9 (17.9-44.6)	33.7 (21.7-54.4)	0.23	0.19
Vitamin D deficiency (<50nmol/L)	314 (85.3)	38 (86.4)	276 (85.2)	0.29	0.20

Pre-pregnancy BMI, Pre-pregnancy body mass index; SBP, systolic blood pressure; DBP, diastolic blood pressure; FBG, fasting blood glucose; HbA1c, hemoglobin A1c or glycated hemoglobin; HDL, high-density lipoprotein; LDL, low-density lipoprotein; Data presented as mean ± SD and median (25th–75th) percentiles for Gaussian and non-Gaussian variables. *P-value adjusted for age and pre-pregnancy BMI; significant at 0.05 and 0.01.

### FokI VDR Gene Polymorphisms and MetS Risk

The disparity between the two groups in the presence of genotypes in *FokI* was discovered to be statistically significant even after adjustment for age (**Table 2**). The frequencies of genotypes *FF*, *Ff*, and *ff* in the control group were 62.7%, 31.4%, and 5.9%, respectively, while for women with MetS, those numbers were 50%, 36.4%, and 13.6%, respectively. The genotype *ff* is a risk factor for MetS (OR = 4.17; 95% CI, 1.42-12.2, *p* = 0.009). The *f* allele is also a genetic risk factor for MetS in this population, given that the prevalence of *F* and *f* alleles for the *FokI* VDR polymorphisms in the two groups was statistically significant (allele *F* vs. *f*; *p* = 0.017).

**Table 2:** Genotype distribution of VDR *FokI* polymorphisms in MetS and control groups.

FokI Genotype	All	MetS	Control	β	OR (95% CI)	*P-Value	Adjusted OR	**P-Value
FF	225 (61.1)	22 (50.0)	203 (62.7)		1		1	
F*f*	118 (32.1)	16 (36.4)	102 (31.4)	0.37	1.45 (0.73-2.88)	0.29	1.42 (0.71-2.9)	0.31
*ff*	25 (6.8)	6 (13.6)	19 (5.9)	1.07	2.91 (1.05-8.1)	0.04	4.17 (1.42-12.2)	0.009
F*f*+*ff*	143 (38.9)	22 (50.0)	121 (37.3)	0.52	1.68 (0.89-3.16)	0.11	1.76 (0.92-3.36)	0.09
F	568 (77.2)	60 (68.2)	508 (78.4)		1	0.03	1	0.02
*f*	168 (22.8)	28 (31.8)	140 (21.6)	0.53	1.69 (1.04-2.75)		1.84 (1.05-1.14)	

OR, odds ratio (95% CI); *P-value significant at < 0.05, **P-value adjusted for age and significant at < 0.05, 0.01.

### Clinical Characteristics of the FokI VDR Gene Polymorphisms in MetS and Control Groups

**Table 3** shows the clinical variables that were observed in MetS participants and pregnant controls according to the *FokI* VDR genotype. Those without MetS with the *FF* genotype had a higher pre-pregnancy and current BMI than those with either the F*f* or *ff* genotypes (*p-values* 0.02, 0.03, respectively). There was no association between individual components of MetS and the VDR genotype in participants.

**Table 3:** Clinical characteristics of *FokI* VDR gene polymorphisms in MetS and control groups.

Parameters	FF	F*f*	*ff*	*P*-value
MetS				
N	22	16	6	
Age (years)	29.1 ± 5.4	28.5 ± 5.3	26.8 ± 3.6	0.19
Pre-pregnancy BMI (kg/m^2^)	34.2 ± 4.8	33.3 ± 2.6	30.3 ± 4.3	0.13
Current BMI (kg/m^2^)	35.3 ± 4.8	34.8 ± 3.1	32.6 ± 6.3	0.43
Parity	2.0 (1.0-5.0)	3.0 (1.0-7.0)	4.0 (2.0-6.0)	0.64
Systolic BP (mmHg)	111.9 ± 12.3	112.9 ± 11.4	112.3 ± 14.2	0.87
Diastolic BP (mmHg)	65.6 ± 8.3	67.2 ± 9.1	65.3 ± 7.9	0.47
Hba1c (%)	4.7 ± 0.4	4.8 ± 0.6	4.8 ± 0.4	0.64
Fasting Glucose (mmol/L)	4.5 ± 0.9	4.4 ± 0.7	4.5 ± 0.3	0.59
HOMA-IR	3.7 (1.9-5.3)	3.7 (1.7-5.9)	2.1 (1.1-2.7)	0.13
Insulin (uU/ml)	7.4 (4.1-12.3)	6.4 (4.2-10.9)	6.4 (3.6-17.9)	0.91
HDL-Cholesterol (mmol/L)	1.6 ± 0.4	1.5 ± 0.4	1.6 ± 0.4	0.95
LDL-Cholesterol (mmol/L)	3.8 ± 1.3	3.9 ± 1.2	3.7 ± 1.0	0.75
Total Cholesterol (mmol/L)	6.2 ± 0.6	6.2 ± 1.4	6.2 ± 1.2	0.99
Triglycerides (mmol/L)	2.7 (2.1-3.2)	2.5 (2.2-3.4)	3.3 (2.2-3.4)	0.76
25(OH)D (nmol/L)	29.9 (18–47)	37.3 (20–61)	22.2 (16–28)	0.23
Control				
N	203	102	19	
Age (years)	32.4 ± 7.1	31.8 ± 6.6	26.3 ± 5.5	0.14
Pre-pregnancy BMI (kg/m^2^)	26.6 ± 5.9	24.7 ± 4.3	25.9 ± 5.7	0.02
Current BMI (kg/m^2^)	27.9 ± 6.3	25.9 ± 4.7	27.0 ± 5.9	0.03
Parity	2.0 (1.0-4.0)	2.0 (1.0-3.0)	1.0 (1.0-2.0)	0.44
Systolic BP (mmHg)	120.1 ± 15.3	119.3 ± 15.5	112.0 ± 22.5	0.72
Diastolic BP (mmHg)	70.8 ± 11.6	73.4 ± 10.3	69.7 ± 18.5	0.80
Hba1c (%)	5.1 ± 0.6	5.0 ± 0.8	5.2 ± 0.9	0.84
Fasting Glucose (mmol/L)	5.4 ± 1.5	6.2 ± 1.6	5.0 ± 0.9	0.16
HOMA-IR	1.4 (0.8-2.3)	1.2 (0.8-2.1)	1.3 (0.8-3.6)	0.62
Insulin (uU/ml)	16.2 (9.3-22.7)	15.5 (6.7-24.7)	7.9 (6.0-11.7)	0.08
HDL-Cholesterol (mmol/L)	1.3 ± 0.5	1.4 ± 0.5	1.2 ± 0.2	0.80
LDL-Cholesterol (mmol/L)	4.1 ± 1.6	3.7 ± 1.2	4.1 ± 0.9	0.74
Total Cholesterol (mmol/L)	6.4 ± 1.3	6.4 ± 1.4	6.6 ± 0.9	0.92
Triglycerides (mmol/L)	1.7 (1.3-2.2)	1.7 (1.2-2.2)	1.6 (1.4-2.3)	0.73
25(OH)D (nmol/L)	34.3 (23-54)	31.4 (19-52)	40.4 (23-85)	0.09

FF denotes normal homozygous, Ff denotes heterozygous and ff denotes homozygous genotypes. Data represent mean ± SD and median (25^th^ and 75^th^) percentile for Gaussian and non-Gaussian variables. P-value significance at 0.05.

### VDR FokI Polymorphism Versus Components of MetS

**Figure 2:**
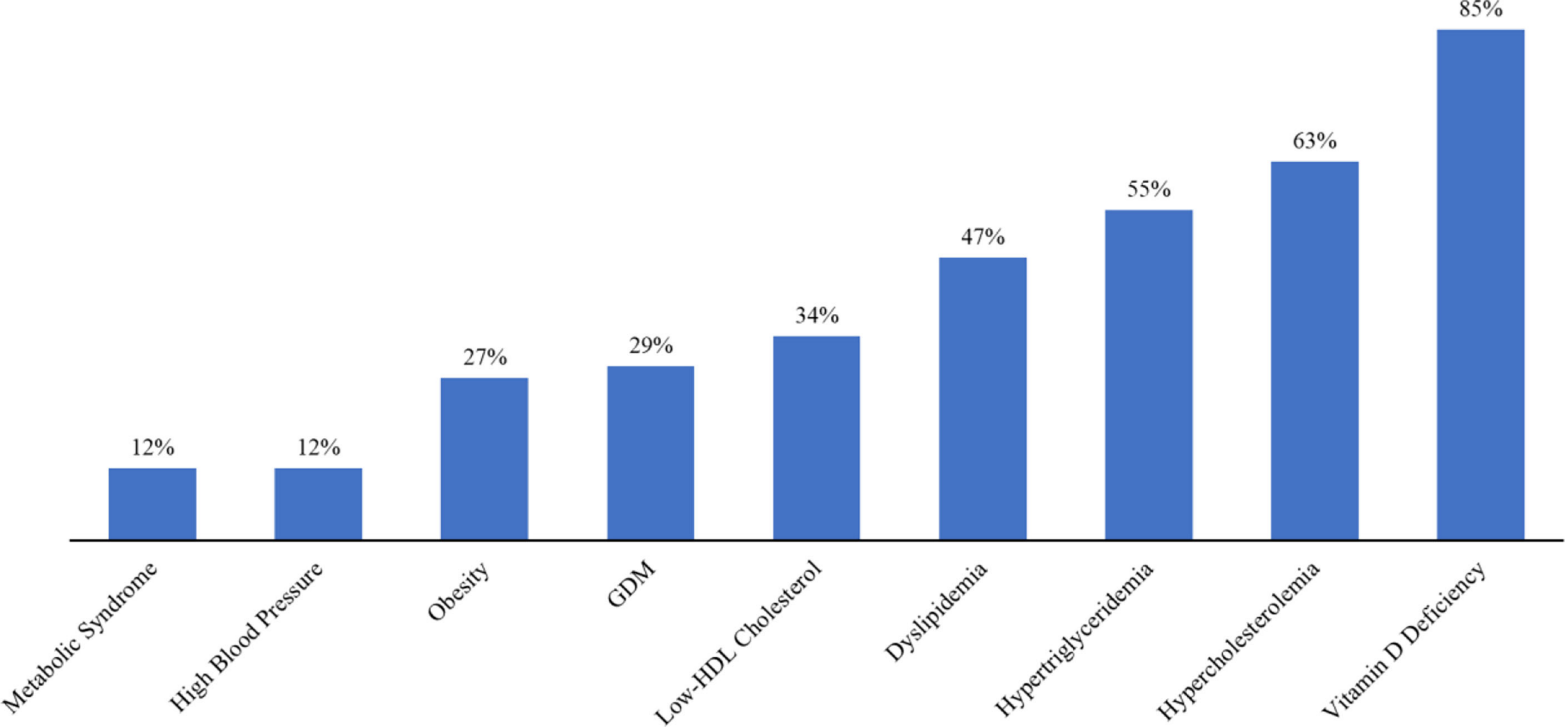
Presence of metabolic disorders among pregnant Saudi women in their first trimester.

**Table 4** shows the multinomial logistic regression between the different genotypes and alleles of *FokI* VDR polymorphisms and obesity, hypertriglyceridemia, low HDL, hypertension, dyslipidemia, hypercholesterolemia, and GDM. The odds ratio for the risk of having any component of MetS was not significant among varying *FokI* VDR genotypes. Participants who had homozygous *ff* genotype were found to have elevated blood pressure (OR = 1.85, 95% CI: 0.79, 4.34), low HDL (OR = 1.14, 95% CI: 0.70, 1.87), dyslipidemia (OR = 1.60, 95% CI: 0.63, 4.10), and GDM (OR = 1.08, 95% CI: 0.66, 1.76). Maternal obesity was present in 27% of participants. Moreover, 12%, 29%, 63%, 55%, 34%, and 47% of participants had elevated blood pressure, GDM, hypercholesterolemia, hypertriglyceridemia, low HDL, and dyslipidemia, respectively (**Figure 2**).

**Table 4:** Risk of the *FokI* VDR gene polymorphisms with MetS and its components.

Parameters	Yes	β	OR (95% CI)	P-Value
BMI ≥ 30 kg/m^2^	101			
FF	66 (65.3)		1	
F*f*	28 (27.4)	0.03	1.03 (0.40-2.63)	0.95
*ff*	7 (7.4)	-0.36	0.70 (0.41-1.18)	0.18
F*f+ff*	35 (34.8)	-0.29	0.75 (0.46-1.22)	0.24
F	160 (28.3)		1	
*F*	42 (25.1)	-0.17	0.84 (0.56-1.27)	0.84
Blood Pressure > 130/85	44			
FF	19 (46.2)		1	
F*f*	19 (46.2)	0.46	1.59 (0.32-7.89)	0.57
*ff*	6 (7.7)	0.62	1.85 (0.79-4.34)	0.16
F*f+ff*	25 (53.9)	0.59	1.81 (0.79-4.11)	0.16
F	57 (10.3)		1	
*F*	31 (14.7)	0.41	1.51 (0.80-2.83)	0.20
HDL-C < 1.3 mmo/L	126			
FF	76 (60.0)		1	
F*f*	42 (33.0)	0.07	1.07 (0.44-2.66)	0.88
*ff*	8 (7.0)	0.13	1.14 (0.70-1.87)	0.59
F*f+ff*	50 (40.0)	0.12	1.13 (0.71-1.79)	0.60
F	194 (33.8)		1	
*F*	58 (35.8)	0.09	1.09 (0.75-1.59)	0.65
Triglycerides > 1.7 mmol/l	202			
FF	127 (62.8)		1	
F*f*	60 (29.6)	0.13	1.14 (0.49-2.65)	0.76
*ff*	15 (7.5)	-0.24	0.79 (0.50-1.23)	0.30
F*f+ff*	75 (37.1)	-0.18	0.84 (0.55-1.28)	0.42
F	314 (55.6)		1	
*F*	90 (53.6)	-0.08	0.92 (0.65-1.31)	0.66
Total Cholesterol > 5.7 mmol/l	233			
FF	137 (58.5)		1	
F*f*	78 (33.6)	0.50	1.65 (0.66-4.12)	0.28
*ff*	18 (7.9)	0.24	1.27 (0.79-2.03)	0.32
F*f+ff*	96 (41.5)	0.28	1.33 (0.85-2.07)	0.21
F	352 (62.1)		1	
*F*	114 (68.1)	0.26	1.31 (0.90-1.89)	0.16
Dyslipidemia	171			
FF	88 (51.5)		1	
F*f*	69 (40.5)	-0.004	1.00 (0.57-1.75)	0.32
*ff*	14 (8.0)	0.47	1.60 (0.63-4.10)	0.99
F*f+ff*	83 (48.5)	0.09	1.09 (0.65-1.84)	0.74
F	245 (71.6)		1	
*F*	97 (29.4)	0.10	1.11 (0.72-1.71)	0.65
GDM	108			
FF	65 (60.2)		1	
F*f*	36 (33.3)	-0.04	0.96 (0.38-2.40)	0.93
*ff*	7 (6.5)	0.06	1.08 (0.66-1.76)	0.76
F*f+ff*	43 (39.8)	0.06	1.06 (0.66-1.68)	0.81
F	166 (76.9)		1	
*F*	50 (23.1)	0.03	1.03 (0.70-1.50)	0.70

FF denotes normal homozygous, Ff denotes heterozygous and ff denotes homozygous genotypes. Data represent unstandardized β, odds ratio, and (95% CI). The P-value is significant at < 0.05.

### MetS and Its Components Versus Vitamin D Status

**Figure 3:**
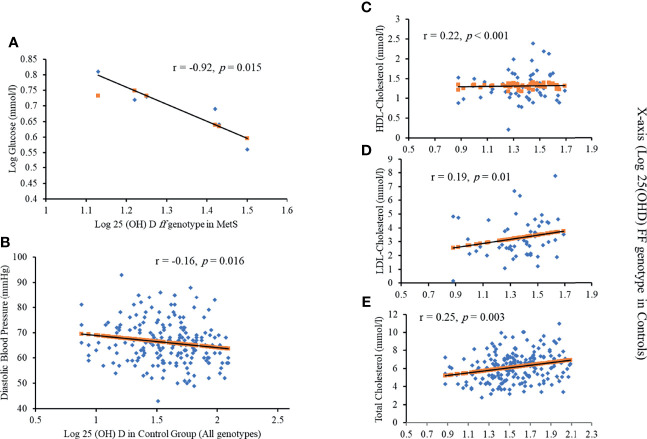
Significant correlations between Log 25(OH)D and **(A)** Log glucose in *ff* genotype in MetS; **(B)** diastolic blood pressure in all genotypes; **(C)** HDL cholesterol in FF genotype in controls; **(D)** LDL cholesterol in FF genotype in controls and **(E)** Total cholesterol in FF genotype in controls

Differences in MetS and control groups in terms of anthropometric and biochemical characteristics were presented in **Supplementary Table 1**. Vitamin D deficiency was present in 70.7% of the participants. Those participants also had higher fasting insulin, HOMA-IR, and systolic blood pressure (SBP) (*p* = 0.007, 0.02, 0.03, respectively). For participants who were not deficient in vitamin D, HDL-C and TC were significantly higher (*p* = 0.001 and 0.049, respectively). Furthermore, those with the *ff* genotype and MetS have a significant and inverse association between serum 25(OH)D and fasting serum glucose (r = -0.92) (**Figure 3A**). A more significant inverse association between serum 25(OH)D and diastolic blood pressure was found in controls, regardless of the genotype (r = -0.16; p<0.05) (**Figure 3B**). Those who carried the *FF* genotype demonstrated a strong association between 25(OH)D, HDL-cholesterol, LDL-cholesterol, and total cholesterol (r = 0.22, 0.19, 0.25, respectively) (**Figures 3C–E**).

## Discussion

The present study is the first evaluation of the links between *FokI* VDR gene polymorphisms and MetS among Saudi Arabian pregnant women. *FokI* VDR polymorphisms may exert a strong impact on MetS susceptibility. It appears that the *f* allele and the *ff* genotype could be risk factors for MetS, whereas the *F* allele and the *FF* genotype could play a protective role. These findings correspond with those of Aslani et al. on postpartum Iranian women with MetS and found that the frequency of the *ff* genotype was two-fold when compared to healthy participants in a postpartum follow-up exercise (OR = 2.1; 95% CI: 0.90–4.89; *p* = 0.09) (21). The same population did not discover significant links between the *FokI* VDR polymorphism and MetS in adult males or females (30). This finding proves to be consistent with additional studies conducted in China (3), the United Arab Emirates (UAE) (18) and Brazil (2). The differences between the studies could be attributed to a range of environmental differences experienced by the populations (31). Likewise, the difference in findings in the Iranian studies demonstrates that there is a substantial difference between ethnic groups in terms of the frequency of the VDR *FokI* genotypes and alleles—even with identical population samples (18).

The association between *FokI* VDR polymorphisms and obesity has been uncovered in a previous study. Mackawy and Badawi (19) found that, in Egypt, the *ff* genotype had a link with higher waist circumference in diabetic patients with MetS when compared to the FF genotype in the same participants. Zaki et al. (16) discovered that obese Egyptian women with a vitamin D deficiency exhibited higher frequencies of the mutant allele *f* when compared to healthy obese women who registered a healthy level of vitamin D. Haris and Baig noted the potential of a link between the *ff* genotype and obesity in their preliminary study on obese Pakistanis (32). Zhao et al. (3) supported this finding, discovering that the *FF* genotype bears a link with lower BMI in Chinese adults with MetS than other genotypes. A significant association between the *f* allele and pre-pregnancy obesity was observed in pregnant Iranian women by Aslani et al. (21). In this study, the control-group participants who carried the *FF* genotype exhibited a higher pre-pregnancy BMI than those who carried F*f* and *ff* genotypes. While not deemed significant, this finding may indicate that the *ff* genotype could be protective against obesity.

The study also discovered a significant inverse correlation between serum 25(OH)D and diastolic blood pressure across all genotypes in non-MetS participants. Those who carried the mutant genotype *ff* also had a higher propensity for hypertension than homozygous and heterozygous genotypes. These results though not significant reflect Zaki et al.’s (16) study that Egyptian women with genotypes *Ff* and *ff* demonstrate higher blood pressure than those who had the more common homozygous genotype *FF*. Two additional studies uncovered a strong link between the *FF* genotype and a risk of high blood pressure. The first studied Emirati women and linked the *FF* genotype to higher systolic blood pressure (18), and the second study uncovered a link between the *FF* genotype and hypertension among Indian adults (15). Both studies attributed elevated blood pressure or hypertension to enhanced production of renin and angiotensin II due to the effect of *FF* homozygotes. Cottone et al. (33) explored hypertensive Italian participants and did not detect a significant link between the *FokI* VDR polymorphism and blood pressure or between blood pressure and serum 25(OH)D levels. Future research may demonstrate a strong link between *FokI* VDR polymorphisms and maternal hypertension. Until then, it is important to understand that when compared to healthy pregnant women, those with hypertension have an increased risk of premature birth and other complications, such as a defect in endothelial-dependent vascular function (4).

Many studies have uncovered a link between the VDR gene polymorphism and dyslipidemia, supporting this relationship (18, 19, 33, 34). Schuch et al. (2) found, for example, that participants who did not suffer from MetS and carried the mutant homozygous genotype (*ff*) exhibited significantly higher TG levels and lower HDL levels when compared to study participants who possessed the heterozygous (F*f*) or normal homozygous (*FF*) genotypes. Reciprocal outcomes have been reached by a host of other researchers. Mackawy and Badawi (19) observed, for example, that when compared to carriers of the *FF* genotype, diabetic, non-MetS carriers of the *ff* genotype exhibited higher plasma TC, TG, and LDL-C with lower HDL-C levels. Hasan et al. (18) studied Emirati carriers of the *ff* genotype and found that when compared to other genotypes of the *FokI* VDR polymorphism, they exhibited higher serum TC levels. These results are consistent with those of this study, which found that when compared to participants carrying the normal homozygous and heterozygous genotypes, those with the mutant, homozygous genotype exhibited a higher risk of developing dyslipidemia. These results are not, however, statistically significant. Moreover, all participants with MetS—regardless of genotype—exhibited a significant association between HDL and serum 25(OH)D levels. In all non-MetS participants carrying *FF* genotypes, serum 25(OH)D levels were found to be significantly associated with HDL, LDL, and TC. Surprisingly, it was discovered that compared to participants with vitamin D deficiency, non-deficient women exhibited a higher lipid profile, similar to the study of Al-Ajlan et al. (10), which discovered that serum vitamin D levels were significantly and positively correlated with serum TG and TC levels in a subgroup of pregnant Saudi women with a vitamin D deficiency. Researchers have speculated that the cause for this outcome could be a link between the high metabolic demands of pregnancy and vitamin D deficiency (10). An increase in lipid levels is a normal physiological consequence of pregnancy, but it is important to understand that by disrupting normal placentation and damaging the vessel wall *via* increased oxidative stress, maternal hyperlipidemia can lead to a host of serious health issues in the fetus (6). Previous research has discovered a strong link between preterm birth and hyperlipidemia, a leading cause of prenatal morbidity and mortality (35, 36).

Many researchers were inspired to explore the link between VDR gene polymorphisms and glucose homeostasis due to the presence of VDR in human tissue and pancreatic β-cells (18, 21). In Brazil, for example, Schuch et al. (2) recorded a significantly higher level of β-cell secretion (HOMA-β) in individuals carrying the *f* allele when compared to participants carrying the F allele. In Brazilian and Egyptian populations, respectively, Schuch et al. (2) and Zaki et al. (18) discovered an important link between the *ff* genotype and a higher HOMA-IR when compared to the *FF* and *Ff* genotypes of the *FokI* VDR polymorphism. Mackawy et al. (21) also discovered that Egyptian patients who carried the *ff* genotype, were diabetic, and suffered from MetS exhibited higher insulin levels and HOMA-IR than those with the *FF* and *Ff* genotypes. These results support our findings of a statistically significant and inverse correlation between serum 25(OH)D levels and fasting serum glucose in pregnant women carrying the *ff* genotype in the MetS group.

Very few studies have explored the effect of the *FokI* VDR polymorphism on GDM. Contradictory results were achieved in a pair of studies exploring Iranian (21) and Saudi populations (24), which explored the relationship between the *FokI* VDR polymorphism and GDM risk. In Iran, Aslani et al. (21) discovered that the frequency of the *ff* genotype in GDM patients was higher than in normal pregnant women (10.6% vs. 6.2%). Healthy participants were also discovered to exhibit a higher frequency of the *F* allele, leading to a suggestion that the *F* allele could play a role as a protective factor against GDM (30). El-Beshbishy et al. (24) conducted their research in Saudi Arabia and found that when compared to their GDM group, there was a higher frequency of the F allele (56.4% vs. 35.7%) and a lower frequency of the *f* allele (43.6% vs. 64.3%) in their control group. However, these results were not statistically significant (*P* = 0.100). The authors of this research concluded that no significant association existed between the *FokI* VDR polymorphism and GDM in the Saudi population (24). The results from this study support this assertion, given that we did not uncover a statistically significant link between GDM and the *FokI* VDR polymorphism. In logistic regression analysis, participants with the *ff* genotype are at higher risk of developing GDM compared to carriers of other genotypes of the *FokI* VDR polymorphism (odds ratio = 1.27; 95% CI: 0.76–2.14; *P* = 0.360, after adjustments for age and BMI). It is widely accepted that while both conditions cause defects in insulin secretory response, *GDM* mimics T2DM in its pathology. We posit that a larger cohort study that had the necessary statistical power would affirm the potential of VDR genetic variation in predicting GDM.

This study had its limitations. A limited frequency of the mutant *ff* genotype meant that we were unable to record a link between *FokI* and components of MetS. For prospective studies, we believe it is important that sufficient statistical power is present, particularly when it comes to the study of the effect of the mutant *ff FokI* genotype on MetS and its components. The study also analyzed the VDR gene at only one SNP, though this SNP was one that translates structurally diverse VDR proteins, which vary in their potential to elicit vitamin D-mediated gene expression. Unfortunately, the study did not accommodate a range of factors that may impact the risk of MetS, such as dietary habits, physical activity, or other lifestyle factors. Despite these limitations, this study is the first to explore the link between the *FokI* VDR polymorphism and MetS and its components in pregnant women from Saudi Arabia. To our knowledge, no previous study has explored these associations in pregnancy among any population. The accuracy of the vitamin D measurements can be assured, given that we explored the role of vitamin D status and correlated it with different variables related to the components of MetS in all *FokI* genotypes. In this study, unlike previous epidemiological studies, precise measurement was used for each MetS component. Blood pressure and the fasting serum samples—including the lipid, glucose, and insulin concentrations—were precisely measured and not self-reported.

## Conclusion

Our study uncovered a statistically significant association between the *FokI* VDR polymorphism and an increased risk of MetS, particularly in participants who carried the *ff* genotype. This link could be used as a prognostic tool to predict maternal MetS risk in Saudi women. This study proposes that the *FokI* VDR polymorphism could have an impact upon key components of MetS, including obesity, dyslipidemia, and GDM. While the link is not statistically significant, our findings should be investigated further in larger cohorts, and additional associative studies should be conducted on other VDR gene polymorphisms.

## Data Availability Statement

The original contributions presented in the study are included in the article/**Supplementary Material**. Further inquiries can be directed to the corresponding author.

## Ethics Statement

The studies involving human participants were reviewed and approved by Ethics Committee of the College of Science, King Saud University, Riyadh, Saudi Arabia. The patients/participants provided their written informed consent to participate in this study.

## Author Contributions

MA, NA-D, and RW contributed in study conception and design. SA, MF, AA-A, and DB dealt in recruitment of participants and procurement of samples. SS and AM analyzed samples and data and MA wrote the manuscript. SS reviewed the manuscript. MA and NA-D supervised the study. All authors contributed to the article and approved the submitted version.

## Funding

The authors are grateful to the Deanship of Scientific Research, King Saud University for funding this research project through Vice Deanship of Scientific Research Chairs.

## Conflict of Interest

The authors declare that the research was conducted in the absence of any commercial or financial relationships that could be construed as a potential conflict of interest.

## Publisher’s Note

All claims expressed in this article are solely those of the authors and do not necessarily represent those of their affiliated organizations, or those of the publisher, the editors and the reviewers. Any product that may be evaluated in this article, or claim that may be made by its manufacturer, is not guaranteed or endorsed by the publisher.
