# Corrigendum: The association between FokI vitamin D receptor polymorphisms with metabolic syndrome among pregnant Arab women

**DOI:** 10.3389/fendo.2023.1200121

**Published:** 2023-04-24

**Authors:** Maysa Alzaim, Nasser M. Al-Daghri, Shaun Sabico, Mona A. Fouda, Sara Al-Musharaf, Malak N. K. Khattak, Abdul Khader Mohammed, Abdulrahman Al-Ajlan, Dalal N. Binjawhar, Richard Wood

**Affiliations:** ^1^ Nutrition Department School of Public Health & Health Sciences, University of Massachusetts, Amherst, MA, United States; ^2^ Biochemistry Department, College of Science, King Saud University, Riyadh, Saudi Arabia; ^3^ Endocrinology Division, Department of Medicine, College of Medicine, King Saud University, Riyadh, Saudi Arabia; ^4^ Department of Community Health, College of Applied Medical Science, King Saud University, Riyadh, Saudi Arabia; ^5^ Sharjah Institute for Medical Research, University of Sharjah, Sharjah, United Arab Emirates; ^6^ Department of Clinical Lab Sciences, College of Applied Medical Sciences, King Saud University, Riyadh, Saudi Arabia; ^7^ Department of Chemistry, College of Science, Princess Nourah bint Abdulrahman University, Riyadh, Saudi Arabia

**Keywords:** metabolic syndrome, vitamin D polymorphisms, pregnancy, gestational diabetes mellitus, genetic marker

**Incorrect Affiliation**

In the published article, there was an error in affiliation 7. Instead of “Department of Chemistry, College of Science, Princess Noura bint Abdulrahman University, Riyadh, Saudi Arabia”, it should be “Department of Chemistry, College of Science, Princess Nourah bint Abdulrahman University, Riyadh, Saudi Arabia”.

The authors apologize for this error and state that this does not change the scientific conclusions of the article in any way. The original article has been updated.

