# Supplementary material for: The Association Between FokI Vitamin D Receptor Polymorphisms With Metabolic Syndrome Among Pregnant Arab Women
Source: Front Endocrinol (Lausanne). 2022 Feb 24;13:844472. doi: 10.3389/fendo.2022.844472 (PMC8909137; doi:10.3389/fendo.2022.844472)
Supplement: Supplementary file 1 [file Table_1.docx]

Supplementary Material

**Supplementary Table 1.** Clinical characteristics of the vitamin D deficient vs. non-deficient pregnant Saudi women.

| **Parameters** | **25(OH)D < 50 nmol/l** | **25(OH)D > 50 nmol/l** | **P-value** |
| --- | --- | --- | --- |
| N | 260 (70.7) | 108 (29.3) |  |
| **Age (years)** | 28.9±5.7 | 29.4±5.2 | 0.47 |
| **Pre-pregnancy BMI** (kg/m^2^) | 26.8±6.1 | 26.9±5.5 | 0.85 |
| **Current BMI** (kg/m^2^) | 28.2±6.5 | 28.1±5.6 | 0.84 |
| **Parity** | 2.0 (1.0-4.0) | 1.0 (1.0-3.0) | 0.20 |
| **Systolic BP (mmHg)** | 114.6±12.9 | 110.8±12.4 | 0.03 |
| **Diastolic BP (mmHg)** | 67.7±9.1 | 65.5±9.2 | 0.07 |
| HbA_1c_ (%) | 6.3 (5.3-7.4) | 6.8 (5.6-9.1) | 0.76 |
| **Fasting Glucose (**mmol/L) | 4.5 (4.1-4.9) | 4.2 (3.9-5.1) | 0.08 |
| **HOMA-IR** | 7.6 (4.6-13.1) | 6.3 (4.5-12.9) | 0.02 |
| **Insulin** (uU/ml) | 1.5 (0.9-2.5) | 1.3 (0.9-2.7) | 0.007 |
| **HDL-Cholesterol** (mmol/L) | 1.5±0.4 | 1.6±0.4 | 0.001 |
| **LDL-Cholesterol** (mmol/L) | 3.8±1.3 | 3.9±1.3 | 0.67 |
| **Total Cholesterol** (mmol/L) | 6.1±1.5 | 6.4±1.4 | 0.049 |
| **Triglycerides** (mmol/L) | 1.80 (1.4-2.3) | 1.90 (1.5-2.4) | 0.68 |
| **25(OH)D** (nmol/L) | 29.9 (20.1-46.7) | 66.7 (43.4-85.3) | <0.001 |

Note: Data represent mean ± SD and median (25^th^ and 75^th^) percentile for Gaussian and non-Gaussian variables. *P*-value denotes significance at *P* < 0.05 and 0.01.
